# Predicting the need for diabetic macular oedema treatment from photographic screening in the Singapore Integrated Diabetic Retinopathy Programme (SiDRP)

**DOI:** 10.1038/s41433-025-03725-1

**Published:** 2025-02-28

**Authors:** Stanley S. J. Poh, Kelvin Y. C. Teo, Rose Ann Goh, Qian Xin Lee, Haslina Hamzah, Serene S. C. Sim, Colin S. Tan, Ngiap Chuan Tan, Tien Yin Wong, Gavin S. W. Tan

**Affiliations:** 1 Singapore National Eye Centre, Singapore, Singapore; 2 Singapore Eye Research Institute, Singapore, Singapore; 3 SNEC Ocular Reading Centre, Singapore National Eye Centre, Singapore, Singapore; 4 Duke-NUS Medical School, National University of Singapore, Ophthalmology and Visual Sciences Academic Clinical Programme, Singapore, Singapore; 5 Ophthalmology, Tan Tock Seng Hospital, National Healthcare Group Eye Institute, Singapore, Singapore; 6 Outram Polyclinic, SingHealth Polyclinics, Singapore, Singapore; 7 Tsinghua Medicine, Tsinghua University, Beijing, People’s Republic of China; 8 School of Clinical Medicine, Beijing Tsinghua Changgung Hospital, Beijing, People’s Republic of China

**Keywords:** Risk factors, Prognostic markers

## Abstract

**Objective:**

To identify diabetic maculopathy features from photographic screening that are predictive of treatment on referral to a tertiary care centre.

**Methods:**

Retrospective review of participants who underwent screening by Singapore Integrated Diabetic Retinopathy Programme from 2015 to 2019. Participants underwent visual acuity (VA) test and non-stereoscopic retinal photographs. Maculopathy features include haemorrhages, microaneurysm and hard exudates (HE), stratified by inner and outer zone (1 and 1-2 disc diameter from fovea respectively) and VA of 6/12. Diabetic macular oedema (DMO) treatment was defined as intravitreal injection or macular photocoagulation up to 540 days from point of referral.

**Results:**

16,712 patients screened had referable eye disease. Out of 3518 maculopathy suspects, 281 (8.0%) received DMO treatment within 540 days. Those treated for DMO had shorter duration of diabetes (6.90 vs. 9.13 years, *p* < 0.001), higher total cholesterol (4.65 ± 1.20 vs. 4.36 ± 1.13 mmol/L, *p* = 0.001) and LDL cholesterol (2.59 ± 1.05 vs. 2.37 ± 0.93 mmol/L, *p* < 0.05) than those without treatment. High-risk features, including inner zone haemorrhages with VA ≤ 6/12 (HR 12.0, 95% CI: 5.5–25.9) and inner zone hard exudates (HR 7.4, 95% CI: 3.4–15.8), significantly increased the likelihood of requiring DMO treatment compared to low-risk features. Higher body mass index is protective of DMO treatment in mild non-proliferative diabetic retinopathy (HR 0.84, 95% CI: 0.73–0.97).

**Conclusion:**

Haemorrhages, microaneurysms and HE within inner zone are important photographic features predictive of DMO treatment. VA is an important stratification for screening especially in patients with only visible haemorrhages.

## Introduction

Diabetic retinopathy screening remains a cornerstone in the early detection of sight-threatening microvascular complications of diabetes [1]. While patients with advanced diabetic retinopathy (DR) can still be asymptomatic in the late stages, early or mild diabetic macular oedema (DMO) may be visually symptomatic, especially when it involves the fovea. A recent meta-analysis reported a prevalence of 3.7% for DMO and a mean annual incidence of 0.5% for clinically significant DMO in individuals with type 2 diabetes [2]. In Singapore, a survey of 10,000 participants using digital retinal photography reported a much higher prevalence of 7.6% for DMO and 6.4% for clinically significant DMO [3]. It is therefore important to have a robust screening protocol, as evidence has emerged that prompt DMO treatment with anti-vascular endothelial growth factor (VEGF) can significantly improve vision [4, 5] and has better long-term outcomes compared to delayed treatment [6, 7].

The presence and severity of DMO in clinical care is often defined using optical coherence tomography (OCT). However, DR screening in primary care is typically limited to 2-dimensional fundus photography. This method lacks stereoscopic and cross-sectional viewing and therefore cannot determine true retinal thickening. At present, there is no consensus on the screening criteria for a maculopathy suspect. The International Clinical Diabetic Retinopathy Severity Scale developed by the International Council of Ophthalmology (ICO) recommends screening of DMO using hard exudates (HE) and apparent retinal thickening [8]. The severity can then be further stratified if the examiner has access to stereoscopic photography allowing better quantification of retinal thickening and distance from the fovea. However, the ICO does not provide clear guidance on the exact distance from the fovea on photographic screening that is indicative of centre-involving DMO requiring treatment, nor how urgent these referrals should be.

More recent DMO screening programmes have used additional surrogate markers like microaneurysms and haemorrhages at the macula as indicators of DMO, although the exact criteria still vary significantly between programmes. The Diabetic Eye Screening Programme (DESP) from the UK and the Irish Diabetic RetinaScreen Programme from Ireland use the presence of microaneurysms, dot blot haemorrhages, and HE as surrogates for maculopathy, and referral urgency is further stratified by the distance of lesions from fovea [9–11]. The Scottish Diabetic Retinopathy Screening Service defines referable maculopathy as any blot haemorrhages or macular oedema within 1 disc diameter (DD) of the fovea [12]. It reported that diabetic maculopathy was the most common reason for referral at 73%. More recently, OCT has also been included as a secondary evaluation in DESP to determine true macular oedema in the latest guidance for DMO surveillance published in July 2020 [13].

The efficacy of these screening programmes in identifying cases that require treatment for DMO is not well studied. Current treatment of DMO may depend not just on the presence of DMO but whether it is centre involving, of sufficient severity, and associated with visual impairment [14]. The importance of individual features or combinations of features in predicting the need for treatment is also unclear. Hence, the aims of our study are to (i) evaluate the effectiveness of our national screening programme in identifying eyes with significant DMO requiring treatment and (ii) identify maculopathy features and systemic factors that are predictive of DMO treatment.

## Methodology

This is a retrospective, observational, cohort study of participants who underwent screening by Singapore Integrated Diabetic Retinopathy Programme (SiDRP) from 2015 to 2019 and subsequently referred to Singapore National Eye Centre. This study was conducted according to the tenets of Declaration of Helsinki and was granted approval by SingHealth Centralised Institutional Review Board. SiDRP was set up in 2010 and rolled out to all primary care centres to optimise DR screening [15]. It consisted of a centralised team of non-clinician graders trained and accredited to grade fundus photos. Details of this screening programme have been previously described [16]. Patients with diabetes were automatically enroled and those screened positive for referable DR or DMO were referred promptly to the specialist outpatient clinic for further evaluation. Fundus photos that were ungradable and patients who defaulted clinic appointments were excluded from the final analysis of this study.

### Image acquisition

At the primary care setting, diabetic patients enroled in SiDRP underwent visual acuity (VA) testing and two field 45° non-stereoscopic retinal digital photographs per eye, one centred at the fovea and the other centred at the disc. Using a two-tiered model, primary graders first review the quality of fundal images and grade the fundal images using stringent criteria described below. Features of DR and diabetic maculopathy were graded separately. Images graded as abnormal were diverted to secondary graders for confirmation.

### Definition of diabetic maculopathy suspect

The presence and severity of diabetic maculopathy were defined based on 3 broad categories, (1) the presence of features of diabetic maculopathy: (i) haemorrhages including microaneurysm, dot and blot haemorrhages or (ii) HE at the macula. (2) These features were further categorised based on their distance from the fovea and VA. The inner zone was defined as 1 DD from the fovea while the outer zone was the area within 1 to 2 DD from the fovea. (3) Images with only visible haemorrhages were further stratified by VA, using 6/12 as a cutoff.

### Classification and screening outcomes

Supplementary Table 1 summarises the seven screening outcomes of diabetic maculopathy based on severity. Of note, patients who had only haemorrhages within the inner zone with VA better than 6/12 underwent a repeat DR screening in 6 months and were only referred if these features persisted on repeat screening. The seven maculopathy features listed here are in order of decreasing severity: High-risk maculopathy features include (i) HE within inner zone and (ii) haemorrhages within inner zone with VA 6/12 or worse. Moderate-risk maculopathy features include (iii) HE within outer zone, (iv) haemorrhages within outer zone with VA 6/12 or worse, (v) haemorrhages within inner zone with VA better than 6/12 (on repeat screening). Low-risk maculopathy features include (vi) haemorrhages within inner zone with VA better than 6/12 and (vii) haemorrhages within outer zone with VA better than 6/12.

Where multiple maculopathy features are present on an image, it will be labelled with the most severe maculopathy feature. If both eyes had features of diabetic maculopathy, the indication of referral will be based on the more severe eye. High- and moderate-risk maculopathy cases are referred to a tertiary institution within 1 and 3 months respectively, whereas low-risk maculopathy cases are non-referable. Images were also concurrently graded for DR status and other conditions such as glaucoma suspect, age-related macular degeneration, retinal vein occlusion, epiretinal membrane, optic disc pathology or significant media opacity if present. The referral for these other conditions would supersede that of diabetic maculopathy if they were deemed more urgent.

### Follow up in tertiary institution

Patients referred to our tertiary centre were divided into two broad groups. The maculopathy suspect arm consists of all features of maculopathy described above. Given that low-risk maculopathy is not referred, this group of patients was included opportunistically when they were referred for other abnormalities on DR screening. The non-maculopathy suspect arm consists of patients who did not have any maculopathy on screening but were referred similarly for other abnormalities. This is also our pseudo-control arm. All patients who were followed up for at least 540 days were included in our study. DMO was confirmed with OCT and treatment with intravitreal anti-VEGF was offered if there was presence of centre involved DMO defined by central subfield thickness of >320 µm on Heidelberg Spectralis OCT (Heidelberg Engineering, Heidelberg, Germany) with significant visual impairment, defined by VA 6/12 or worse. A subset of patients received macular photocoagulation for non-centre involving DMO with clinically significant macular oedema as defined in the Early Treatment of Diabetic Retinopathy Study (ETDRS) study [17], as deemed appropriate by the treating physician. Time to treatment was taken from the day of referral to the day of treatment. Patients were followed up for up to 540 days to account referral delays and ensure at least 360 days of follow-up at the centre.

### Statiscal analysis

All statistical analysis was performed using RStudio v2023.06.0 (RStudio Team, Boston, MA). Categorical variables were expressed as frequency and percentages, and Pearson’s Chi-squared test was used to compare between groups. Continuous variables were expressed as mean ± standard deviation and the Welch Two Sample *t*-test was used to compare between two means that are normally distributed. A *p*-value of <0.05 was considered indicative of a statistically significant difference. A multivariate cox-proportional hazards model was used to assess risk factors associated with DMO treatment. The survival of patients receiving DMO treatment up to 540 days was plotted on a Kaplan-Meier curve.

## Results

**Fig. 1:**
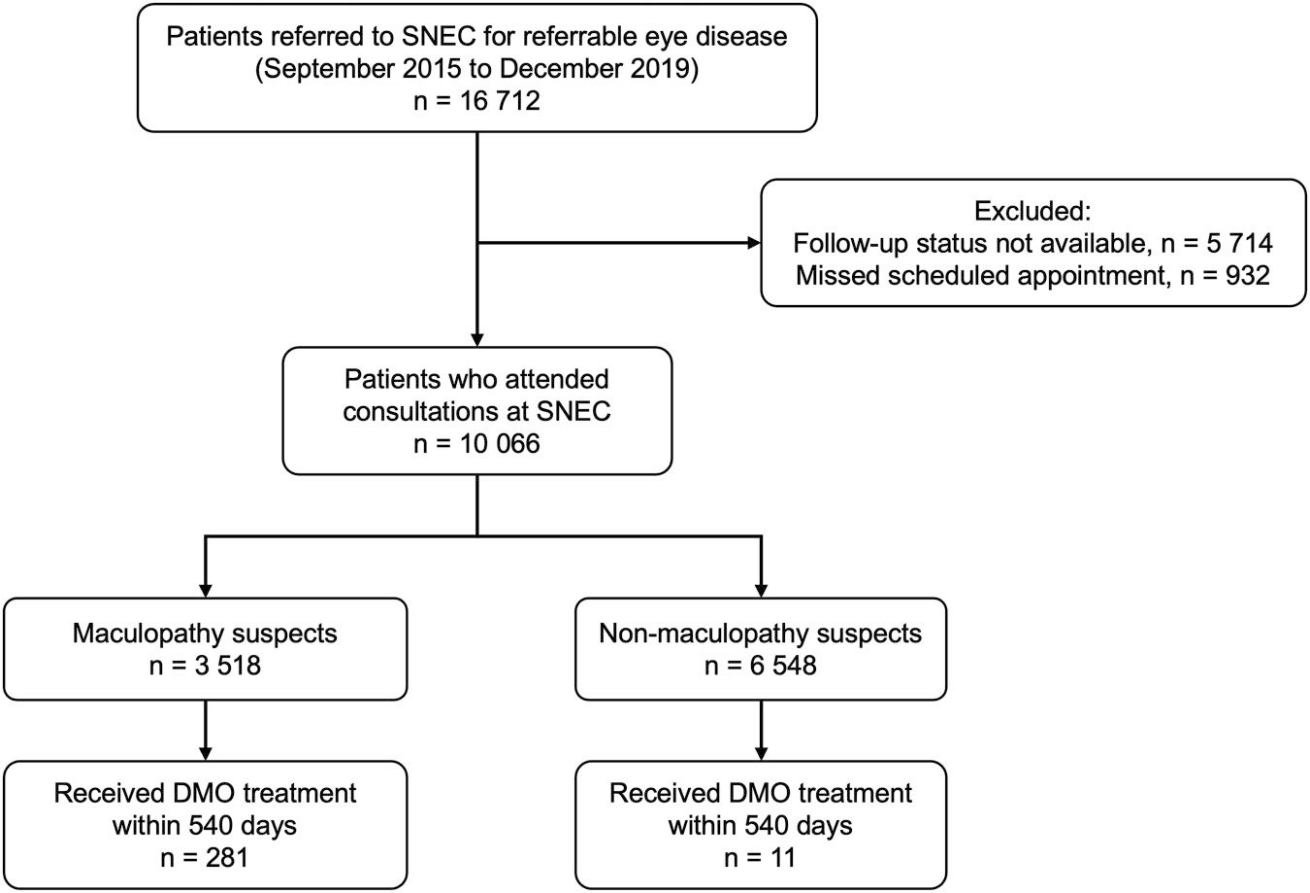
Flow chart illustrating diabetic maculopathy and non-maculopathy suspects who were screened in the Singapore Integrated Diabetic Retinopathy Programme and subsequently referred to Singapore National Eye Centre (SNEC).

Between September 2015 and December 2019, a total of 16,712 patients were referred to the Singapore National Eye Centre (SNEC) from SiDRP. Patients with ungradable maculopathy features and those who defaulted appointments were excluded from this study. The study flow chart is illustrated in Fig. 1. Of the 10,066 patients who attended their scheduled consultations, 3 518 (34.9%) individuals were identified as maculopathy suspects, and 6 548 (65.1%) were non-maculopathy suspects. Within 540 days from time of referral, 281 (8.0%) of the maculopathy suspects received DMO treatment. The non-maculopathy suspects arm consists of patients with other referable conditions described above, of which 11 (0.2%) received DMO treatment.

Table 1 shows the baseline characteristics of maculopathy suspects, comparing those with and without DMO treatment. There was no significant difference in gender and age, but the proportion of ethnicities was significantly different between groups.

**Table 1:** Baseline characteristics of maculopathy suspect group comparing patients with and without treatment for diabetic macular oedema within 540 days.

	DMO treatment (*n* = 281)	No DMO treatment (*n* = 3237)	*p*-value
Male	170 (60%)	1821 (56%)	0.189
Age, years	58.9 ± 9.8	58.1 ± 12.4	0.197
Ethnicity			
Chinese	144 (52%)	2004 (62%)	<0.001
Indian	45 (16%)	518 (16%)	
Malay	72 (25%)	561 (17%)	
Others	20 (7.2%)	154 (4.8%)	
Duration of diabetes, years	6.90 ± 7.07	9.13 ± 7.77	<0.001
HbA1c, %	8.35 ± 2.16	8.23 ± 1.99	0.425
Systolic BP, mmHg	137.7 ± 18.4	134.7 ± 17.2	0.018
Diastolic BP, mmHg	73.4 ± 9.5	72.2 ± 10.1	0.083
Creatinine, µmol/L	88.0 ± 50.3	82.6 ± 49.6	0.132
Total cholesterol, mmol/L	4.65 ± 1.20	4.36 ± 1.13	0.001
HDL cholesterol, mmol/L	1.31 ± 0.34	1.26 ± 0.35	0.122
LDL Cholesterol, mmol/L	2.59 ± 1.05	2.37 ± 0.93	0.004
BMI, kg/m^2^	26.0 ± 4.5	26.6 ± 4.9	0.056

*DMO* diabetic macular oedema, *BP* blood pressure, *HDL* high-density lipoprotein, *LDL* low-density lipoprotein, *BMI* body mass index.

We found that individuals who required DMO treatment had higher blood pressure (137.7 ± 18.4 mmHg vs. 134.7 ± 17.2 mmHg, *p* < 0.018), total cholesterol (4.65 ± 1.20 mmol/L vs. 4.36 ± 1.13 mmol/L, *p* = 0.001) and low-density lipoprotein (LDL) cholesterol (2.59 ± 1.05 mmol/L vs. 2.37 ± 0.93 mmol/L, *p* = 0.004) than those who did not require treatment. Those who required treatment also had shorter duration of diabetes compared to those who did not require treatment (6.90 ± 7.07 years vs. 9.13 ± 7.77 years, *p* < 0.001).

Table 2 shows the multivariate analysis of risk factors associated with DMO treatment, adjusted for body mass index, blood pressure, serum creatinine, serum cholesterol and HbA1_c_ levels. No systemic risks were identified as significantly associated with DMO treatment in this cohort. The maculopathy feature with the highest risk of treatment was haemorrhages within inner zone with VA 6/12 or worse (HR 12.0, 95% CI 5.5–25.9), followed by HE within inner zone (HR 7.4, 95% CI 3.3–15.8) when compared to the lowest risk maculopathy feature, haemorrhages within outer zone with VA better than 6/12. On stratification by DR status, proliferative DR (HR 7.63, 95% CI 4.00–14.56), severe non-proliferative DR (HR 10.10, 95% CI 6.00–17.02) and moderate non-proliferative DR (HR 5.42, 95% CI 3.27–8.99) were all significantly more likely to be treated for DMO when compared to mild non-proliferative DR.

**Table 2:** Multivariate analysis of systemic and ocular features associated with diabetic macular oedema treatment.

	HR	95% CI	*p*-value
**Systemic features**			
BMI, kg/m2	0.99	0.96–1.02	0.390
Systolic blood pressure, mmHg	1.00	1.00–1.01	0.542
Diastolic blood pressure, mmHg	1.00	0.99–1.02	0.686
Creatinine, μmol/L	1.00	1.00–1.00	0.223
LDL cholesterol, mmol/L	1.03	0.90–1.18	0.646
HbA1c, %	0.95	0.89–1.01	0.104
**Diabetic maculopathy feature**			
Haemorrhages within inner zone with VA 6/12 or worse	12.00	5.54–25.95	<0.001
Hard exudates within inner zone	7.36	3.44–15.75	<0.001
Hard exudates within outer zone	1.98	0.75–5.22	0.165
Haemorrhages within outer zone with VA 6/12 or worse	1.29	0.33–5.01	0.712
Haemorrhages within inner zone with VA better than 6/12	0.58	0.22–1.55	0.278
Haemorrhages within inner zone with VA better than 6/12 (on repeat screening)	0.50	0.06–4.18	0.525
Haemorrhages within outer zone with VA better than 6/12*	1.00		
**Diabetic retinopathy status**			
Proliferative DR	7.63	4.00–14.56	<0.001
Severe non-proliferative DR	10.10	6.00–17.02	<0.001
Moderate non-proliferative DR	5.42	3.27–8.99	<0.001
Mild non-proliferative DR*	1.00		

*HR* Hazard ratio, *CI* confidence interval, *BMI* body mass index, *LDL* low-density lipoprotein, *VA* visual acuity, *DR* diabetic retinopathy

*Reference group.

In the subgroup analysis of the cohort with mild non-proliferative DR, higher body mass index was found to be a protective factor of DMO treatment (HR 0.84, 95% CI: 0.73–0.97) (Table 3). No other systemic factors were found to be significant in this subgroup. We also did not identify any specific maculopathy feature that was more likely to be treated for DMO in this subgroup.

**Table 3:** Subgroup analysis of systemic association of eyes with mild non-proliferative diabetic retinopathy requiring diabetic macular oedema treatment.

Systemic features	HR	95% CI	*p*-value
BMI, kg/m2	0.84	0.73–0.97	0.021
Systolic blood pressure, mmHg	1.01	0.98–1.05	0.469
Diastolic blood pressure, mmHg	0.96	0.9–1.02	0.177
Creatinine, μmol/L	1.00	1.00–1.01	0.250
LDL cholesterol, mmol/L	1.18	0.67–2.07	0.575
HbA1c, %	0.78	0.51–1.19	0.252

*HR* Hazard ratio, *CI* confidence interval, *BMI* body mass index, *LDL* low-density lipoprotein.

**Fig. 2:**
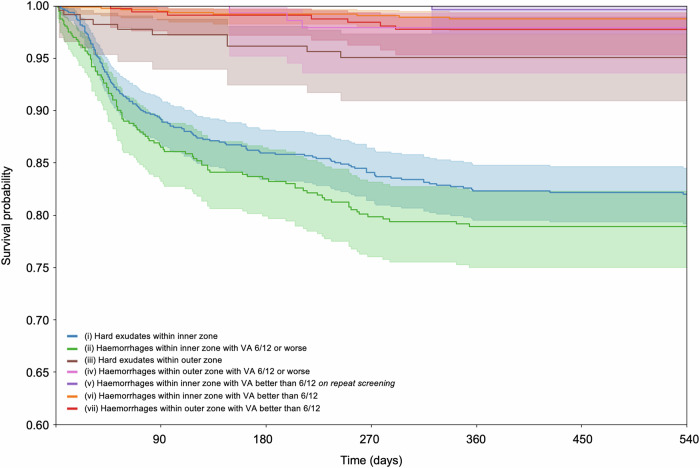
Kaplan-Meier Curve of Time from Referral to Diabetic Macular Oedema Treatment.

Figure 2 illustrates the Kaplan-Meier survival curve from the time of referral to DMO treatment up to 540 days. Over 90% of DMO treatment occurred within 360 days of referral. The two high-risk maculopathy features, haemorrhages within inner zone with VA 6/12 or worse labelled in green and hard exudates within inner zone labelled in blue, showed the most rapid drop in survival in the first 90 days. Approximately 15% of patients in each group received treatment in the first 3 months. These two survival curves continued to drop steadily before stabilising at approximately 270 days where 20–25% of patients in each group had received DMO treatment. There is a significant difference in survival when comparing high-risk to low or moderate-risk maculopathy features (*p* < 0.001) where majority of low to moderate-risk maculopathy feature eyes did not require treatment up to 540 days. The flattening of survival curves beyond 360 days suggests that if the eye did not receive treatment in the first year of referral, it is unlikely to require treatment later on.

## Discussion

Among all referable eye diseases referred to our centre, 34.9% were maculopathy suspects, and of those, 8.0% received treatment for DMO within 540 days. Of the other 65.1% who were non-maculopathy suspects, 0.2% were found to receive DMO treatment in the same timeframe. In the DMO treatment group, 89.3% of patients had high-risk maculopathy features defined by HE within inner zone or haemorrhages within inner zone with VA 6/12 or worse on screening. The remaining 10% of patients who received DMO treatment had low to moderate risk maculopathy features, with lesions further away from the fovea and better vision at the time of referral. The survival curve indicated that this group experienced a longer interval from referral to treatment, likely due to the inherently lower-risk nature of their maculopathy or a less urgent referral process. Patients in the treatment group were also found to have significantly shorter duration of diabetes, higher systolic blood pressure, and higher total and LDL cholesterol levels. The two high-risk maculopathy features were (1) microaneurysms, dot or blot haemorrhages within inner zone with VA 6/12 or worse being 12 times more likely to be treated, and (2) HE within inner zone being 7.4 times more likely to be treated when compared to the low-risk feature.

To our knowledge, our study is the first to report the treatment outcome of maculopathy suspects referral in a national DR screening programme. To account for delays between referral and clinic consultation, we set a 540-day study endpoint to ensure follow-up of at least one year for patients with lower-risk maculopathy identified opportunistically. This timeframe simulates patients without referable disease at their first DR screening who might develop maculopathy by the next annual screening. The first 180 days capture cases requiring immediate or early DMO treatment, while the 360-day interval includes those developing significant DMO within a year. The Kaplan Meier survival curve showed that the highest risk of treatment was in the first 180 days, where almost 20% with high-risk maculopathy required treatment, after which the risk decreased significantly. In a real-world setting, patients who screened negative for maculopathy would not typically be seen at a tertiary centre. Therefore, we used a pseudo-control group consisting of patients referred to our centre for other referable eye diseases but did not exhibit features of maculopathy at the time of screening. In this group, only 0.2% of patients received DMO treatment within the same timeframe. This low false negative rate is reassuring and indicates that the SiDRP screening programme was effective in identifying features predictive of treatment.

An effective photographic screening protocol is important given that OCT and widefield imaging are not widely available as screening tool even in a high-resource setting like Singapore [18]. The SiDRP protocol has undergone several modifications and now includes photographic evidence of microaneurysms, dot blot haemorrhages and HE, further stratified by their distance from the fovea and VA to determine the urgency of referral. In this study, the proportion of maculopathy suspects was 34.9%. This rate is significantly lower than the 73% reported in Scotland in 2010, although it is unclear whether their higher percentage reflects cases of true DMO requiring treatment [12]. The referral criteria in the Scottish Diabetic Retinopathy Service included the presence of haemorrhages, any HE and macular oedema within 1 DD from the fovea, while VA was not used as a screening criterion. In SiDRP, patients with haemorrhages near the fovea without exudates were observed and rescreened in 6 months if vision was good. Further stratification with VA may reduce excessive referral to the tertiary centre. We compared the screening criteria of the two programmes and estimated that, if the Scottish criteria were applied to our cohort, 4% of patients requiring DMO treatment might be missed (Supplementary Table 2).

In the latest UK guideline, high-risk maculopathy was defined as the presence of circinate exudates greater than 1/2 disc area within 1 DD of the fovea and VA of 6/12 or worse. These patients warrant a hospital service referral within 13 weeks [13]. Adopting these criteria for our patient cohort would result in 9% of patients with DMO not being referred, and consequently, not receiving treatment (Supplementary Table 2). The UK also incorporated OCT in this latest guideline where patients with low-risk maculopathy would receive OCT surveillance within 13 weeks and be subsequently referred to the hospital if OCT screened positive. Such screening strategy has proven to improve cost savings although secondary OCT screening may potentially lead to delays in treatment [19]. It is also argued that photographic screening alone generates high false positive referrals and may pose a financial toll on the healthcare system and unnecessary psychological stress for patients [20]. As the cost of OCT continues to decrease, it could potentially be utilised as a secondary screening tool in Singapore for patients with low-risk maculopathy features, following the UK model.

In our study, we found that patients in the DMO treatment group had significantly higher total and LDL cholesterol, although they were not associated with an increased likelihood of DMO treatment on multivariate analysis. Dyslipidaemia is a well-established association with DMO but treatment of dyslipidaemia may not necessarily reduce the risk of DMO [21–23]. It was interesting that despite comparable HbA1c levels, the duration of diabetes was significantly shorter in the treatment group (6.9 years vs. 9.1 years). HbA1c values reported in this study were taken from measurements closest to the point of referral and hence were only a reflection of glycaemic control at referral. It does not provide information on the historical long term diabetes control. The Scottish screening programme found that individuals referred for maculopathy had shorter duration of diabetes when compared to those referred for retinopathy [12]. We postulate that this group of patients may have had poorer initial control of diabetes, leading to earlier and more severe endothelial damage [24].

Overall, our study did not identify any systemic factors that significantly influenced the likelihood of DMO treatment. The study population predominantly comprised patients referred for diabetic maculopathy. Those with referable DR, such as moderate non-proliferative DR or worse but without significant maculopathy, were not included in this cohort and may have influenced the findings. In addition, multiple studies have demonstrated that HbA1c is not a useful predictor of visual loss in DMO [14, 25]. Despite this limitation, maintaining optimal glycaemic control remains critical in slowing the progression of diabetic retinopathy [26, 27]. In a subset of patients with mild non-proliferative DR, higher BMI appeared to be protective. This aligns with reports suggesting that obesity may act as a protective factor against DR and DMO, particularly in Asian populations, a phenomenon referred to as the “obesity paradox” [28–31]. Weight loss is also a metabolic complication of obesity and could indicate more advanced diabetes, complicating this relationship. Moreover, BMI reflects general obesity but does not specifically measure truncal obesity, which is more strongly associated with metabolic syndrome. Given this conflicting evidence, no clear recommendations on weight management can be made at this time.

Our study has some limitations. Firstly, the results may not be representative of the entire population as a significant proportion of patients screened by SiDRP were referred to other institutions. There may be geographical variations in patient demographics and differences in treatment preferences between institutions. Additionally, patients who were not referred might develop DMO and remain asymptomatic and thus would not be captured within our study timeframe. Second, there is an inherent bias in interpreting the Kaplan-Meier survival curve where patients with high-risk features were referred earlier and subsequently received treatment earlier than others. Third, we did not compare the outcomes of photographic screening with OCT-confirmed centre or non-centre-involving DMO using OCT, as we believe that treatment endpoint is more clinically relevant. Treatment decisions are dependent on additional factors such as VA, extent of retinal thickening, DR severity, other ocular comorbidities, and to some extent, physician or patient preference.

## Conclusion

In conclusion, our study comprehensively examines the association between individual maculopathy features and the need for DMO treatment. High-risk features, such as HE and haemorrhages within the inner zone, combined with VA 6/12 or worse on fundus photography, are strong predictors of DMO treatment. VA serves as a valuable criterion to identify patients with only visible haemorrhages but no HE, warranting early referral to a tertiary centre. This approach can help reduce unnecessary referrals. Additionally, our study demonstrates that even patients with lesions located up to 2 DD away from the fovea, may develop significant DMO requiring treatment within 540 days. Photographic screening remains the most cost-effective screening strategy, but it is essential to strike a balance between excessive referrals and the risks of under-referring and under-treating DMO by continually evaluating and improving our screening strategies.

## Summary

### What was known before

- Screening for DMO traditionally relies on fundus photography, which lacks the ability to accurately assess retinal thickness compared to OCT
- There is no standardised screening protocol globally, and different programmes use varied criteria for referral.
- While maculopathy features such as hard exudates, microaneurysms, and haemorrhages are indicators of DMO, their specific impact on treatment likelihood was not well studied

### What this study adds

- High-risk maculopathy features such as hard exudates within inner zone and haemorrhages within the inner zone with VA 6/12 or worse are predictive of DMO treatment
- Visual acuity is a useful parameter to further stratify urgency of referrals for maculopathy suspects
- Higher BMI appears protective in mild non-proliferative DR cases

## Supplementary information

Supplementary Table 1

Supplementary Table 2

## Data Availability

The datasets generated during and/or analysed during the current study are available from the corresponding author on reasonable request.
