# Supplementary material for: Predicting the need for diabetic macular oedema treatment from photographic screening in the Singapore Integrated Diabetic Retinopathy Programme (SiDRP)
Source: Eye (Lond). 2025 Feb 28;39(9):1710–6. doi: 10.1038/s41433-025-03725-1 (PMC12130208; doi:10.1038/s41433-025-03725-1)
Supplement: Supplementary file 1 — Supplementary Table 1 [file 41433_2025_3725_MOESM1_ESM.docx]

Supplementary Table 1. The screening outcomes of Singapore Integrated Diabetic Retinopathy Programme based on individual maculopathy feature

| **Maculopathy feature** | | **Screening outcome** |
| --- | --- | --- |
| High-risk features | (i) Hard exudates within inner zone | Refer in 1 month |
|  | (ii) Haemorrhages within inner zone with VA 6/12 or worse |  |
| Moderate-risk features | (iii) Hard exudates within outer zone | Refer in 3 months |
|  | (iv) Haemorrhages within outer zone with VA 6/12 or worse |  |
|  | (v) Haemorrhages within inner zone with VA better than 6/12 *on repeat screening* |  |
| Low-risk features | (vi) Haemorrhages within inner zone with VA better than 6/12 | Repeat screening in 6 months |
|  | (vii) Haemorrhages within outer zone with VA better than 6/12 | Repeat screening in 1 year |
| VA, visual acuity  Inner zone defined as the area within 1 disc diameter from fovea Outer zone defined as the area within 1 to 2 disc diameter from fovea Haemorrhages include microaneurysms, dot and blot haemorrhages | | |
