# Supplementary material for: Predicting the need for diabetic macular oedema treatment from photographic screening in the Singapore Integrated Diabetic Retinopathy Programme (SiDRP)
Source: Eye (Lond). 2025 Feb 28;39(9):1710–6. doi: 10.1038/s41433-025-03725-1 (PMC12130208; doi:10.1038/s41433-025-03725-1)
Supplement: Supplementary file 2 — Supplementary Table 2 [file 41433_2025_3725_MOESM2_ESM.docx]

Supplementary Table 2. Comparison of screening outcomes of Singapore Integrated Diabetic Retinopathy Programme versus other national screening programmes using the same study population

| **Maculopathy feature** | **Singapore*** | | **United Kingdom** | | | **Scotland** | | |
| --- | --- | --- | --- | --- | --- | --- | --- | --- |
|  | Referred | Treated | | Referred | Treated | | Referred | Treated |
| (i) Hard exudates within inner zone | 343 | 1 | | - | - | | 343 | 1 |
| (ii) Haemorrhages within inner zone with VA 6/12 or worse | 462 | 104 | | 462 | 104 | | 462 | 104 |
| (iii) Hard exudates within outer zone | 857 | 9 | | - | - | | 857 | 9 |
| (iv) Haemorrhages within outer zone with VA 6/12 or worse | 181 | 3 | | 181 | 3 | | - | - |
| (v) Haemorrhages within inner zone with VA better than 6/12 on repeat screening | 363 | 7 | | - | - | | - | - |
| (vi) Haemorrhages within inner zone with VA better than 6/12 | 807 | 147 | | 807 | 147 | | 807 | 147 |
| (vii) Haemorrhages within outer zone with VA better than 6/12 | 224 | 10 | | - | - | | 224 | 10 |
| Number of potentially untreated DMO patients |  |  | | 27 (9.6%) | | | 10 (3.6%) | |
| *Used as reference  VA, visual acuity; DMO, diabetic macular oedema  Inner zone defined as the area within 1 disc diameter from fovea Outer zone defined as the area within 1 to 2 disc diameter from fovea Haemorrhages include microaneurysms, dot and blot haemorrhages | | | | | | | | |
